# Chemometric Methods—A Valuable Tool for Investigating the Interactions Between Antifungal Drugs (Including Antifungal Antibiotics) and Food

**DOI:** 10.3390/antibiotics14010070

**Published:** 2025-01-10

**Authors:** Agnieszka Wiesner-Kiełczewska, Paweł Zagrodzki, Alicja Gawalska, Paweł Paśko

**Affiliations:** 1 Doctoral School of Medical and Health Sciences, Jagiellonian University Medical College, 31-008 Cracow, Poland; agnieszka.wiesner@doctoral.uj.edu.pl; 2 Department of Food Chemistry and Nutrition, Faculty of Pharmacy, Jagiellonian University Medical College, 31-008 Cracow, Poland; pawel.zagrodzki@uj.edu.pl; 3 Department of Medicinal Chemistry, Faculty of Pharmacy, Jagiellonian University Medical College, 31-008 Cracow, Poland; alicja.gawalska@alumni.uj.edu.pl

**Keywords:** antifungal antibiotics, antifungals, chemometrics, partial least square, food, interaction, food–drug interaction, antimicrobial resistance

## Abstract

Background/Objectives: Developing antifungal drugs with lower potential for interactions with food may help to optimize treatment and reduce the risk of antimicrobial resistance. Chemometrics uses statistical and mathematical methods to analyze multivariate chemical data, enabling the identification of key correlations and simplifying data interpretation. We used the partial least squares (PLS) approach to explore the correlations between various characteristics of oral antifungal drugs (including antifungal antibiotics) and dietary interventions, aiming to identify patterns that could inform the optimization of antifungal therapy. Methods: We analyzed 15 oral antifungal drugs, including azoles (8), antifungal antibiotics (4), antifungal antimetabolites (1), squalene epoxidase inhibitors (1), and glucan synthase inhibitors (1). The input dataset comprised information from published clinical trials, chemical records, and calculations. We constructed PLS models with changes in the pharmacokinetic parameters (∆AUC, area under the curve; ∆C_max_, maximum drug concentration; and ∆T_max_, time to reach maximum drug concentration) after dietary intervention as the response parameters and eight groups of molecular descriptors (M1–M8) as the predictor parameters. We performed separate analyses for the different nutritional interventions. Results: In the final PLS model with food as an intervention, we effectively reduced the dimensionality of the dataset while retaining a substantial percentage of the original information (variance), as significant components explained 69.8% and 17.5% of the predictor and response parameter variances, respectively. The PLS model was significant because its components met the cross-validation criteria. We obtained six significant positive and negative correlations between the descriptors related to atoms and the postprandial ∆T_max_. Conclusions: The PLS method is valuable for investigating interactions between antifungal drugs (including antifungal antibiotics) and food. The correlations obtained can be used in drug modeling to predict interactions with dietary interventions based on the antifungal drug’s chemical structure. Incorporating chemometric techniques into the early drug development stages could facilitate the design of antifungal antibiotics and other antifungal agents with optimized absorption in the presence of dietary components.

## 1. Introduction

The World Health Organization (WHO) has identified antimicrobial resistance (AMR) as one of the top ten threats to global public health [1]. Fungi are susceptible to developing resistant strains owing to their rapid reproduction and genetic variability [2]. Among the pathogenic fungi, *Candida krusei*, *Candida auris*, *Candida parapsilosis*, and *Aspergillus fumigatus* pose the greatest concerns [3]. Notably, *C. auris* exhibits alarming resistance levels: approximately 90% of isolates are resistant to fluconazole, 30% to amphotericin B, and 10% to echinocandins [3].

The limited number of oral antifungal drugs available in different pharmacological groups further hinders the implementation of effective treatments. Resistance to a single drug class can severely restrict treatment options, whereas multidrug resistance may render infections untreatable [4]. Diagnosing invasive fungal infections presents additional challenges, particularly in low- and middle-income countries, where diagnostic tools are scarce and antifungal susceptibility testing is rarely available [5]. Delayed or inappropriate treatment due to diagnostic limitations may also increase the risk of resistance [5].

Prolonged antifungal therapies, such as six weeks or longer, e.g., for invasive aspergillosis, are sometimes prescribed to ensure complete eradication of the infection [6]. However, evidence suggests that extending the treatment duration does not necessarily improve patient outcomes [7]. Instead, prolonged use may contribute to the emergence of AMR, particularly in immunocompromised individuals, and facilitate the development of secondary opportunistic infections [7]. Additionally, extended therapy increases the risk of adverse effects and complicates adherence, further undermining the treatment efficacy [8].

Food–drug interactions represent another underexplored yet significant factor that may affect antifungal pharmacotherapy. Our initial literature search indicated that food can substantially influence the bioavailability of more than 60% of antifungals, usually with a high positive effect. Regarding the impact of beverages, milk enhanced griseofulvin absorption, whereas acidic drinks such as Coca-Cola improved the bioavailability of itraconazole, ketoconazole, and posaconazole [9,10,11,12,13,14]. Conversely, orange juice decreased itraconazole absorption [15]. Antacids significantly reduced itraconazole and ketoconazole bioavailability; however, administering posaconazole with nutritional supplements markedly improved its absorption [16,17,18,19]. Due to the abovementioned interactions, developing antifungal drugs with lower potential for interactions with food is essential to optimize treatment and reduce the risk of AMR.

**Figure 1:**
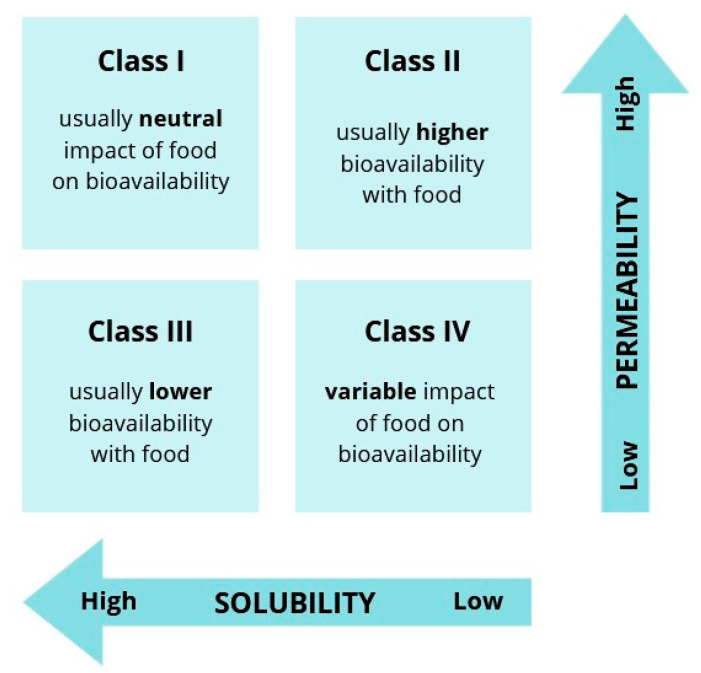
Effect of food on drug bioavailability according to BCS class. Adopted from [21]. Created in https://www.canva.com/ (accessed on 2 December 2024).

The different effects of dietary interventions on the bioavailability of antifungal drugs can partially be explained by their diverse physicochemical properties and chemical structures. The Biopharmaceutical Classification System (BCS) divides medicines into four classes based on their permeability through intestinal barriers and solubility in water [20]. BCS class I compounds are usually well absorbed due to high solubility and permeability. The solvation rate limits the absorption of BCS class II representatives, which are highly permeable but poorly soluble in water. Members of BCS class III efficiently dissolve in water; however, their absorption can be suboptimal due to the low permeability. BCS class IV drugs are generally poorly absorbed because their solubility and permeability are low. The general effect of food (especially high-fat food) on drug pharmacokinetics according to the BCS class is presented in Figure 1 [21].

As outlined in Table 1, most oral antifungal drugs belong to BCS class II (highly permeable and poorly soluble in water), but single members of other classes are also present. Gu et al. developed a logistic regression model to predict how food influences drug absorption, incorporating BCS classification and dosage as factors. However, the analysis included only two antifungal agents, griseofulvin and itraconazole [22]. Another important physicochemical parameter is the logarithm of the partition coefficient (log *p*). The partition coefficient (*p*) quantifies the distribution of an uncharged compound between two immiscible solvents: a lipophilic solvent (e.g., octanol) and a hydrophilic solvent (e.g., water). Drugs with higher log *p* values exhibit greater lipophilicity, and their absorption often improves when taken with high-fat meals due to the increased secretion of bile, micelle formation, and delayed gastric emptying [20]. In contrast, the absorption of hydrophilic compounds (with log *p* values < 1) can be lower in the presence of food rich in fat. As outlined in Table 1, antifungal drugs are mostly highly or moderately lipophilic with single hydrophilic compounds (fluconazole and flucytosine). Some antifungal antibiotics (amphotericin B, nystatin, and hachimycin) possess amphiphilic properties and contain both lipophilic (conjugated polyene chain) and hydrophilic (hydroxyl groups) regions in their structures. By mechanisms similar to lipophilic compounds, high-fat foods may potentially enhance the absorption of amphiphilic drugs, but the extent of this enhancement is highly molecule-dependent.

Our recent research indicates that modern chemometric techniques offer a promising approach for investigating the complex relationships between antiretroviral drug structures, physicochemical features, and pharmacokinetics in the presence of food [23]. Chemometrics uses statistical and mathematical methods to analyze multivariate chemical data, enabling the identification of key correlations and simplifying data interpretation [24].

The partial least squares (PLS) method is one of the most widely utilized chemometric techniques. It is a supervised quantitative chemometric method for predictive modeling and dimensionality reduction in multivariate datasets [25]. PLS evaluates the relationships between predictor and response parameters by creating latent variables—linear combinations of the original parameters—that are optimized to maximize their covariance [26]. PLS effectively handles collinear and non-normally distributed data, making it particularly valuable for pharmaceutical and food-related research [25].

In the present study, we used the PLS method to explore the correlations between various characteristics of oral antifungal drugs (including antifungal antibiotics) and dietary interventions, including food, beverages, antacids, and mineral supplements. We aimed to identify patterns that could inform the optimization of antifungal therapies.

## 2. Results

**Figure 2:**
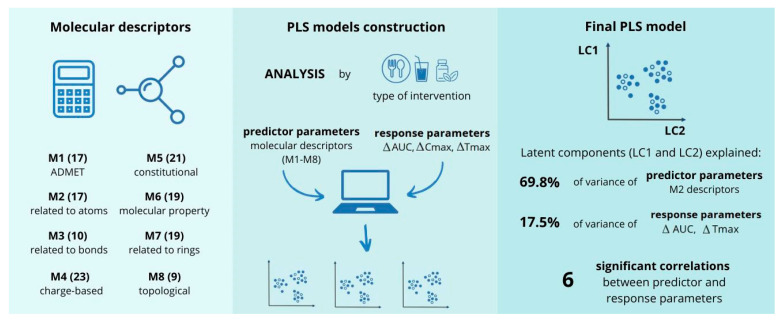
Flowchart of the chemometric analysis of antifungal drugs. Created https://www.canva.com/ (accessed on 3 December 2024).

The general course of the chemometric analysis is shown in Figure 2.

### 2.1. Drugs Under Analysis

Of the 15 antifungal antibiotics and other antifungal agents initially chosen for analysis, we found no studies investigating the impact of dietary interventions on four, namely, albaconazole, amphotericin B, hachimycin (trichomycin), and nystatin. Additionally, one study was available for flucytosine, with only the AUC reported. We included the remaining ten drugs in the chemometric analysis.

### 2.2. Molecular Descriptors Included in the Analysis

We calculated 2532 molecular descriptors using the software tools described in the Section 4. We excluded the parameters with the least variability, and of the remaining 1825, we deleted duplicates and excluded complex electrotopological and charge-based descriptors. The remaining 135 molecular descriptors, classified into eight groups, served as predictor parameters for constructing the PLS models (Figure 2). For a detailed list of the individual molecular descriptors within each group and their definitions, refer to Table A1 in Appendix A.

### 2.3. Analysis Involving Food

We identified 54 studies that investigated the interactions between antifungal drugs and food. We excluded 11 studies with pre- and postprandial values of less than two of the three main pharmacokinetic parameters (AUC, C_max_, and T_max_) reported. Table A2 in Appendix A describes the studies in detail, and Supplementary Material S1 provides the complete final input database.

Only one PLS model reached statistical significance, with the M2 molecular descriptors (related to atoms) serving as the predictor parameters. For all the remaining molecular descriptor groups, correlations between the original parameters and PLS components were insufficient to construct statistically significant and informative PLS models. ∆T_max_ strongly contributed to the model among the response parameters, with absolute loadings exceeding 0.3. We also included the ∆AUC in the model; however, its loadings were low, suggesting that the parameter played a minimal role in defining the latent components of the PLS model. Another response parameter, ∆C_max_, was considered uninformative and excluded from the model because it had even lower loadings on both latent components, meaning it was not significantly correlated with the other parameters.

Table 2 presents the correlation coefficients for the correlated parameter pairs based on the PLS model.

**Figure 3:**
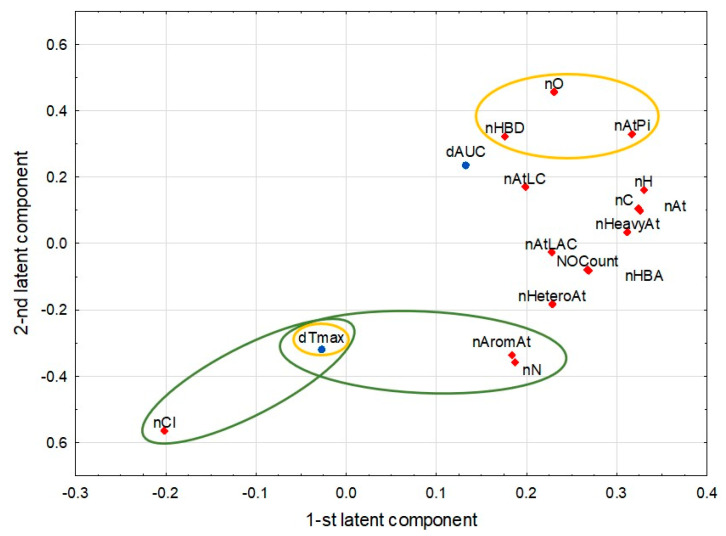
Loadings of the first and second latent components in the PLS model combining the response pharmacokinetic parameters (**blue dots**) with a group of molecular descriptors related to atoms (predictor parameters, **red diamonds**). The strongest positive correlations between the response and predictor parameters are shown in **green ellipses**, whereas the negative ones are shown in **yellow ellipses**. Created in Statistica v.13.3 (licensed software).

The PLS model consisted of two significant components, with eigenvalues of 8.96 and 1.52. Both met the R2 cross-validation rule in the SIMCA software, which states that at least one response variable in the PLS model had a fraction of the variability, which could be predicted by a particular latent component greater than the significance cutoff (0.05). The model explained 69.8% of the variance in the predictor parameters and 17.5% in the response parameters. Figure 3 presents a scatter plot illustrating the weights that link the predictor (blue dots) and response (red diamonds) parameters to the first two latent components in the PLS model. The plot highlights the correlation structure among individual parameters and potential parameter clusters. Parameters near the plot’s edges, with absolute values exceeding 0.3 for either latent component, exert the strongest influence on those components and display greater discriminative power. Clusters of such parametersreflect strong positive correlations, whereas those on opposite sides of the coordinate center exhibit negative correlations.

In the PLS model, we observed the strongest positive correlation coefficients between the response and predictor parameters for ∆T_max_ and the molecular descriptors nCl, nN, and nAromAt, all showing high positive loadings on the second latent component. In contrast, the highest negative correlation coefficients for ∆T_max_ were with a cluster of three strongly correlated molecular descriptors (nO, nHBD, and nAtPi) due to their high negative loadings on the second latent component.

### 2.4. Analyses Involving Antacids/Supplements and Beverages

For antacids, mineral supplements, and beverages, none of the molecular descriptor groups exhibited sufficiently strong correlations with the ∆AUC, ∆C_max_, or ∆T_max_ to allow for the construction of statistically significant and informative PLS models.

## 3. Discussion

### 3.1. Benefits from the Use of Chemometric Methods

The input dataset for building the final PLS model encompassed observations from 43 studies from 40 reports of 10 drugs, 17 predictors, and two response parameters. Identifying the general patterns and correlations in such a complex dataset is challenging because of the numerous, often interrelated, variables and observations. Using the PLS method, we effectively reduced the complexity and dimensionality of the dataset while retaining a substantial percentage of the original information (variance). The significant components of the PLS model accounted for 69.8% of the variance in the predictor parameters. The explained variance was lower (17.5%) for the response parameters but still sufficient to observe and reliably interpret the correlations. Additionally, the chemometric approach enabled us to visualize the relationships and patterns within the analyzed datasets on a relatively easy-to-interpret two-dimensional plot (Figure 3).

### 3.2. Interpretation of Correlations

Most studies with specified meal types involved high-fat or standard meals. Hence, during the interpretation, we assumed that the obtained correlations refer to standard/high-fat meals. We focused on interpreting correlations between the predictor and response parameters only (highlighted in blue in Table 2), as these were most relevant to our research aims.

We observed a positive correlation between the number of chlorine atoms (nCl) and the rate of drug absorption in the presence of a standard/high-fat meal (∆T_max_). Unlike highly polar functional groups, such as hydroxyl (-OH) or amine (-NH_2_), chlorine does not form strong hydrogen bonds with water, limiting its ability to enhance water solubility. Instead, the relatively large atomic radius and hydrophobic surface of chlorine increase the affinity of a molecule for lipids [27]. Chlorine atoms withdraw electron density from adjacent structures, stabilizing the molecule but not promoting water attraction. These electron-poor nonpolar regions are more soluble in lipids, further enhancing lipophilicity with each additional chlorine atom [27]. Among the analyzed antifungal antibiotics and other antifungal agents, chlorine atoms are present in itraconazole, ketoconazole, and griseofulvin. These drugs exhibit moderate to high lipophilicity and belong to the BCS II class, characterized by high membrane permeability and low aqueous solubility. BCS II drugs depend on bile salts and dietary lipids to improve dissolution, particularly in high-fat environments, where they partition into lipid micelles. However, apart from solubilization, antifungals can be trapped in micelles formed by conjugated bile salts, which may slow their release into the gastrointestinal lumen and delay their absorption [28,29]. Furthermore, high-fat meals may also delay gastric emptying, compounding the effect on the T_max_ [30].

The numbers of nitrogen atoms (nNs) and aromatic atoms (nAromAts) exhibited a strong positive correlation between each other and the ∆T_max_. Nitrogen atoms in the structures of antifungal agents are primarily found in the aromatic imidazole and triazole rings and, to a lesser extent, in the aliphatic six-membered rings. Despite the high electronegativity of nitrogen, its incorporation into these flat molecular structures increases the drug’s mass, volume, and overall lipophilicity. Additionally, the lone pair of electrons on nitrogen in aromatic systems participate in electronic delocalization, stabilizing the molecule and potentially supporting its affinity for lipid-rich environments, which facilitates absorption [31]. Therefore, the nitrogen atoms in the heteroaromatic systems of antifungal drugs contribute to the electronic and structural properties of the molecule, enhancing its lipophilicity and stability. These effects may indirectly promote the partitioning of the drug into lipid micelles and its interaction with bile salts in the gastrointestinal environment, aiding solubilization under high-fat conditions. A higher number of aromatic atoms (greater aromaticity) also contributes to molecular lipophilicity due to the delocalized π-electron system in aromatic rings, which provides a nonpolar, hydrophobic surface [32]. Additionally, the planarity and rigidity of aromatic systems may reduce the overall polarity and flexibility of molecules, further reinforcing their hydrophobic character. These structural features allow antifungal drugs to align efficiently with lipid bilayers and micelles, maximizing lipid phase compatibility and reducing aqueous phase solubility. Consequently, the combined impact of nN and nAromAt descriptors may support prolonged interactions within lipid-rich environments, which can delay the T_max_ while optimizing the absorption in the presence of dietary fat.

We observed a negative correlation between the number of hydrogen bond donors (nHBDs) or oxygen atoms (nO) and ∆T_max_. A higher number of hydrogen bond donors in a molecule translates into greater potential to form hydrogen bonds, which improves the molecule’s solubility in water and increases its hydrophilicity [33]. Functional groups, such as hydroxyl (-OH) or amine (-NH_2_), which contribute to the nHBDs and nO, enhance the drug’s interaction with water, facilitating faster dissolution in the aqueous phase and initial absorption. However, the increased hydrophilicity associated with these groups may limit the drug’s passive diffusion across lipid-rich biological membranes, as hydrophilic molecules have a lower affinity for the lipid bilayers of cellular membranes [33]. The intake of a drug with a high-fat meal may partially mitigate this limitation because of the enhanced solubility mechanisms in the fat-rich environment. Despite this assistance, the dependence of hydrophilic antifungals on lipid-mediated mechanisms for absorption is lower than their lipophilic counterparts, which may explain the less pronounced impact of food on their ∆T_max_. Additionally, the rapid dissolution of hydrophilic drugs may offset the slower diffusion through membranes, leading to quicker attainment of peak plasma concentrations.

A negative correlation between the number of atoms in the largest π-system (nAtPi) and ∆T_max_ may appear contradictory, given that increasing aromaticity typically enhances lipophilicity. However, we may explain this apparent contradiction by considering the role of polar heteroatoms within the π-system. Namely, nAtPi also showed a strong positive correlation with the nO and nHBDs, indicating that the large π-systems in antifungal drugs often contain polar atoms. These heteroatoms introduce partial charges and increase molecular polarizability, promoting solubility in the aqueous phase through hydrogen bonding and other dipole–dipole interactions with water molecules. This effect may counterbalance the tendency of the π-system to partition into lipids, leading to faster drug release from the lipid micelles, easier diffusion through biological membranes, and quicker absorption.

Interestingly, in our previous work regarding antiretroviral drugs, we observed the opposite directions of correlations between molecular descriptors related to lipophilicity and ∆T_max_; specifically, with increasing lipophilicity, the rate of postprandial absorption was faster [23]. These conflicting results may arise from substantial differences in the physicochemical properties and chemical structures of antifungal and antiretroviral drugs. Antifungal drugs represent a highly specialized group of compounds with unique physicochemical and pharmacokinetic properties. As such, their behavior may often deviate from general trends observed in other drug classes. Structures of antifungal drugs are generally rigid with multi-ring systems or extended hydrophobic groups. Most of the analyzed antifungals were highly lipophilic (log *p* values > 4) BCS class II drugs, meaning they rely heavily on bile salts and dietary lipids to enhance their dissolution in the gastrointestinal tract. Therefore, food has a positive or neutral effect on the overall bioavailability of most antifungal drugs. In contrast, antiretroviral drugs are more heterogeneous, with representatives of all four BCS classes (the majority belong to BCS II and BCS III classes), and significantly differ in log *p* values (from hydrophilic to highly lipophilic) and chemical structures [20]. The impact of food on the pharmacokinetic parameters of antiretroviral drugs is also diverse and independent of the pharmacological group [20]. Our results indicate that, given the substantial differences between classes of antimicrobial drugs, correlations obtained for one class should not be extrapolated to others.

### 3.3. Limitations of This Study

Several limitations of the input data and the analysis itself support the cautious interpretation and limited generalization of our results.

#### 3.3.1. Quality and Heterogeneity of Input Data

We judged the quality of the studies serving as the data source for chemometric analysis to be relatively poor. 70% of the studies had a moderate or high risk of bias (RoB), whereas only 9% were of good quality (low RoB). We could not judge the RoB of nine studies because of the insufficiently described design (see Table A2 in Appendix A). Only 65% of the studies followed the design recommended by the FDA for assessing the food effect, which is open-label and cross-over. A total of 63% were randomized; however, most lacked sufficient details regarding the randomization process. As we did not restrict study years, some reports were from the 1980s and the 1990s and often provided scarce methodological information, further complicating bias assessment.

Significant gaps in the data were apparent, limiting the applicability of the collected parameters for chemometric analysis. Regarding participant characteristics, gender was left unspecified in 28% of the studies, and race was not reported in more than half (58%) of the cases. In most studies, age was mentioned; however, usually, as a mean or range, both measures were rarely given. Although the type of meal was specified in almost all (91%) studies, qualitative and quantitative meal composition details were both present in only 26% (Table A2 in Appendix A).

Another critical limitation of the input data is their high heterogeneity. Different doses and formulations of antiretroviral drugs were tested. Although all the studies involved healthy adult volunteers, their age, gender, and race differed. A total of 52% of the studies with specified gender included only males, and in the remaining, women were underrepresented. In studies with specified races, the majority were Caucasian, and the minority were African-American (Table A2 in Appendix A). Most studies involved high-fat meals; however, other types, such as standard, low-fat, and high-protein meals, were also present. Even studies involving the same type of meal (e.g., high-fat) often differed in meal composition.

#### 3.3.2. Chemometric Analysis

The major limitation of our study is the uneven representation of the data for individual antifungal drugs. A total of 5 of the 15 drugs initially chosen for the analysis were not included in the PLS model because of the lack of or insufficient data regarding the impact of dietary interventions on PK parameters. Three of these drugs—amphotericin B, hachimycin, and nystatin—are antifungal antibiotics sharing a similar structure (conjugated polyene chain with multiple hydroxyl groups), different from antifungals of the other pharmacological groups. Their exclusion from the analysis resulted in the loss of valuable insight into the potential relationships between the amphoteric properties of the compound and the effects of dietary interventions. However, polyene antifungal antibiotics are rarely administered orally due to their very low bioavailability, which may explain the lack of studies on food effects. Although our analysis covered all the most frequently used oral antifungal drugs, the available evidence on the impact of food was disproportionate, as 31 of the 43 studies included in the analysis concerned two drugs, itraconazole and posaconazole, whereas for several drugs (ibrexafungerp, isavuconazole, oteseconazole, and terbinafine), only one food effect study was available. Consequently, our results should be cautiously generalized to antifungal agents other than those analyzed, particularly if they possess substantially different structures.

The available evidence also varies regarding the impact of different dietary interventions on antifungal pharmacokinetics. Compared to food, the input datasets for analyses involving beverages and antacids/supplements were relatively small, comprising only records from 13 and 7 studies, respectively. This may partially explain why we obtained a statistically significant PLS model solely in the food analysis. Therefore, the correlations obtained in the analysis of food effect studies may not be relevant to other dietary interventions.

We chose molecular descriptors as the predictor parameters to investigate the relationships between drug structure and postprandial PK parameters. We grouped the molecular descriptors into eight groups to limit the number of predictor parameters and constructed a PLS model for each group separately. One of the eight PLS models obtained in this manner reached statistical significance. We also tested another approach: we chose two molecular descriptors from each of the eight groups. The first selection criterion was the highest number of unique values, and the second was the largest standard deviation among the parameters. Subsequently, we constructed a PLS model with molecular descriptors of the highest variability serving as the predictor parameters; however, this model was statistically insignificant.

The PLS model with descriptors related to atoms explained only 17.5% of the variance in the response parameters (∆AUC, ∆C_max_, and ∆T_max_). This substantially limits the predictive power and generalizability of the model. The low value of the explained variance could be primarily attributed to the heterogeneity of the input data. Apart from the drug structure expressed by molecular descriptors, other factors, such as variable study designs, different drug formulations tested, participant characteristics (gender, race, etc.), and meal composition, may substantially contribute to the overall variance of the dataset. We could not include all these factors in the analysis due to the numerous gaps in the data we already discussed and the risk of model overfitting. However, we cannot rule out their possible impact on the effect of interaction with food.

On the other hand, future research should also consider alternative modeling approaches to facilitate clinical outcome prediction. For example, orthogonal projections to the latent structure statistical model (OPLS), instead of the traditional PLS, may significantly improve interpretability, study power, and statistical validity.

## 4. Materials and Methods

### 4.1. Data Collection and Preparation

#### 4.1.1. Drugs Selected for the Analysis

To ensure the global relevance of our study, we aimed to examine all oral antifungal drugs that, to our knowledge, are or were available internationally. We considered the long-established medicines, those introduced to the market recently, and experimental. A total of 15 drugs were categorized according to their pharmacological groups:

Azoles (8)—albaconazole, fluconazole, isavuconazole, itraconazole, ketoconazole, oteseconazole, posaconazole, and voriconazole;
Antifungal antibiotics (4)—amphotericin B, griseofulvin, hachimycin (trichomycin), and nystatin;
Antifungal antimetabolites (1)—flucytosine;
Squalene epoxidase inhibitors (1)—terbinafine;
Glucan synthase inhibitors (1)—ibrexafungerp.

We are aware that amphotericin B and nystatin have very low oral bioavailability. However, both drugs can be administered orally when local action is required, for example, to prevent or treat fungal infections of the gastrointestinal tract [34,35]. Therefore, we did not exclude them from the analysis.

Although ketoconazole is no longer widely approved for treating fungal infections, we included it because it remains authorized in Europe to treat Cushing’s syndrome [36]. Additionally, the FDA permits its use for endemic mycoses when other antifungal therapies are ineffective [37].

#### 4.1.2. Data from Clinical Trials

We searched three databases, Medline (via PubMed), Embase, and the Cochrane Library, for clinical trials examining the effects of dietary interventions (food, beverages, antacids, and mineral supplements) on the pharmacokinetics of oral antifungal antibiotics and other antifungal agents to use them as source data. We used the following keywords: antifungal drug names combined with “food”, “food-drug interaction”, “drug-food interaction”, “fed”, “fasted”, “fasting”, “postprandial”, “meal”, “breakfast”, “dietary supplement”, “antacids”, “milk”, “coffee”, “coca”, “cola”, “coke”, “beverage”, and “juice”. We found additional studies in the summaries of product characteristics (SmPCs) of antifungal drugs available worldwide and the reference lists of studies previously identified. Additionally, we explored the gray literature by performing a targeted search on Google Scholar.

We considered only the original studies involving humans (letters, reviews, in vitro and in silico studies, and those performed on animals were excluded). To possess a comprehensive pool of evidence, we made no other restrictions regarding the study design, language, publication year, sample size, or patient characteristics (health status, race, gender, and age).

To judge the quality of the input data, we performed the risk of bias (RoB) assessment using different tools suitable for the study design (parallel trials—version 2 of the Cochrane RoB tool [38], cross-over studies—RoB 2—additional considerations for cross-over trials [39], and longitudinal studies—the NIH quality assessment tool for before–after (pre–post) studies [40]).

From the collected studies, we extracted information on antifungal agents (including antifungal antibiotics), e.g., their dose, formulation, and pharmacokinetic parameters with and without dietary intervention. We excluded studies that lacked information on the values of at least two of the three key pharmacokinetic parameters (AUC, C_max_, and T_max_) before and after the dietary intervention. The remaining studies were considered for inclusion in the final chemometric models.

Additionally, for studies assessing the influence of food or beverages, we documented details such as meal type (e.g., high-fat, high-protein, and low-fat), beverage type (e.g., milk, juice, and coffee), meal/beverage composition, and quantitative meal characteristics, such as caloric density and macronutrient proportions (fat, carbohydrate, and protein). From studies involving antacids and mineral supplements, we recorded data on their type (e.g., aluminum, magnesium, and iron), dose, and formulation.

We calculated the percentage changes in the pharmacokinetic parameters of antifungal antibiotics and other antifungal drugs (∆AUC, ∆C_max_, and ∆T_max_) in the presence and absence of dietary interventions. These values served as response parameters in the chemometric models.

#### 4.1.3. Data from Chemical Records

We retrieved each drug’s BCS class (if available), predicted water solubility, and protein-binding percentage from chemical databases. DrugBank was searched first, and in the absence of information, other chemical records, such as PubChem and Micromedex, were used.

#### 4.1.4. Molecular Descriptors

Molecular descriptors mathematically characterize the physical and chemical properties of each antifungal drug. We used the following software tools to calculate the molecular descriptors: Admetlab2.0 [41], Chemopy1.0 [42], PaDEL-Descriptor v.2.21 [43], QikProp (Schrodinger 2024-4) [44], and the rdkit.Chem.Descriptors module [45].

We combined the sets of molecular descriptors generated by each software in the MS Excel spreadsheet. Subsequently, we excluded molecular descriptors with minimal variability from the analysis, specifically those with only one or two distinct values across all antifungal drugs. Such parameters do not significantly contribute to explaining the data variance and are unlikely to provide meaningful information for building the PLS model. In the next step, duplicate parameters (generated by more than one software) were manually removed. Additionally, we excluded complex and detailed electrotopological and charge-based molecular descriptors because the biological interpretation of correlations involving these parameters poses challenges.

We grouped the remaining molecular descriptors as follows:

M1: ADMET descriptors: address pharmacokinetic properties such as absorption, distribution, metabolism (e.g., CYP enzymes, glycoprotein P, or OATP substrates and inhibitors), excretion, and toxicity;
M2: descriptors related to atoms: represent the number of specific types of atoms in a drug molecule;
M3: descriptors related to bonds: indicate the number and types of bonds within a drug molecule;
M4: charge-based descriptors: capture the distribution of molecular charges;
M5: constitutional descriptors: reflect the fundamental chemical composition and various aspects of molecular size;
M6: molecular property descriptors: relate to the basic physicochemical properties of the drug molecule;
M7: descriptors related to rings: count and classify different types of rings in the molecular structure;
M8: topological descriptors: describe the molecular shape, branching patterns, and internal atomic arrangement.

### 4.2. Chemometric Analysis

We used SIMCA-P v.9 software to develop PLS models, with changes in the pharmacokinetic parameters (∆AUC, ∆C_max_, and ∆T_max_) as response parameters and the molecular descriptor groups (M1–M8) as predictor parameters.

We performed a PLS analysis after data standardization (z-transformation) to obtain the zero mean and unit variance for each parameter. Therefore, for each data value, the mean of the corresponding parameter was subtracted, and the results were divided by the standard deviation of the same parameter.

Latent components in the PLS models were constructed as linear combinations of the original parameters and were iteratively optimized to maximize alignment with the path model, effectively capturing the variance of both the predictor and response parameters while considering their interrelationships.

Using leave-one-out cross-validation, we determined the optimal number of latent components (model complexity). Each data point was excluded from the model one at a time, and its actual response parameter values were compared to those predicted by the model. This comparison allowed for the calculation of residuals. We repeated this procedure until all data were excluded precisely once. The model with the smallest total sum of squared residuals across all data points was identified as optimal because it demonstrated the greatest predictive accuracy.

We conducted separate analyses for different dietary interventions, including food, beverages, and antacids or mineral supplements.

## 5. Conclusions

During the chemometric analysis, we revealed six significant positive and negative correlations between the antifungal drug structure parameters (molecular descriptors related to atoms) and their absorption rate in the presence of food (expressed as ∆T_max_).

Antifungal drugs (including antifungal antibiotics) represent another class of antimicrobial agents for which we have demonstrated the utility of chemometric methods as valuable tools for studying interactions with dietary interventions. Our findings emphasize the critical role of the chemical structure and physicochemical properties in the design of biologically active molecules to mitigate adverse food–drug interactions. The observed correlations provide a foundation for drug modeling, potentially enabling the prediction of dietary interactions based on the drug’s chemical structure. Incorporating chemometric techniques into the early stages of drug development may facilitate the design of antifungal antibiotics and other antifungal agents with optimized absorption in the presence of dietary components.

Moreover, chemometric approaches, aimed at simplifying complex datasets and uncovering correlations among multiple variables, may offer promising potential for exploring drug–drug interactions in future research. This study also highlights the pressing need for comprehensive databases detailing dietary interaction profiles across a broad spectrum of drugs. Such resources would enhance the development of robust chemometric models, contributing to the creation of safer and more effective therapeutic regimens.

## Figures and Tables

**Table 1:** BCS classification and log *p* values of oral antifungal drugs.

Drug	BCS	Log *p*
Albaconazole	NS	2.82
Amphotericin B	IV	−0.04
Fluconazole	I	0.56
Flucytosine	III	−1.04
Griseofulvin	II	2.17
Hachimycin (trichomycin)	NS	2.39
Ibrexafungerp	IV	7.16
Isavuconazole	II	4.14
Itraconazole	II	7.31
Ketoconazole	II	4.19
Nystatin	IV	0.33
Oteseconazole	II	4.69
Posaconazole	II	5.41
Terbinafine	II	5.53
Voriconazole	II	1.82

**Table 2:** Correlation coefficients for pairs of parameters based on the PLS model (pairs of predictor and response parameters are highlighted in blue).

PLS Model Predictor Parameters—Molecular Descriptors Related to Atoms (M2) Response Parameters—Postprandial ∆AUC and ∆T_max_		
Pairs of Correlated Parameters		Correlation Coefficient
nAromAt	nN	1.000
nAt	nC	1.000
nO	nHBD	0.999
nC	nH	0.991
nAt	nH	0.987
nAt	nHeavyAt	0.982
nC	nHeavyAt	0.978
nCl	∆T_max_	0.967
nAtPi	nHBD	0.965
nAtPi	nO	0.956
nH	nHeavyAt	0.941
nAtPi	nH	0.938
nAtPi	nC	0.882
nAt	nAtPi	0.872
nN	∆T_max_	0.844
nAromAt	∆T_max_	0.835
nAtPi	nHeavyAt	0.765
nCl	nN	0.679
nAromAt	nCl	0.666
nAromAt	nAtPi	−0.299
nAtPi	nN	−0.316
nAromAt	nHBD	−0.539
nN	nHBD	−0.553
nAromAt	nO	−0.566
nN	nO	−0.579
nAtPi	∆T_max_	−0.775
nAtPi	nCl	−0.911
nHBD	∆T_max_	−0.914
nO	∆T_max_	−0.926
nCl	nHBD	−0.987
nCl	nO	−0.992

## Data Availability

The original contributions presented in this study are included in the article/Supplementary Materials. Further inquiries can be directed to the corresponding author(s).

## Supplementary Materials

The following supporting information can be downloaded at https://www.mdpi.com/article/10.3390/antibiotics14010070/s1, Supplementary Material S1—Complete final input database for chemometric analysis.

## Appendix A

**Table A1:** List of molecular descriptors with their explanations.

M1—ADMET Descriptors (17)		
Molecular Descriptor Name	Explanation	Software
CYP1A2(inh)	Probability of being CYP inhibitor	admetlab2.0
CYP1A2(s)	Probability of being CYP substrate	admetlab2.0
CYP2C19(inh)	Probability of being CYP inhibitor	admetlab2.0
CYP2C19(s)	Probability of being CYP substrate	admetlab2.0
CYP2C9(inh)	Probability of being CYP inhibitor	admetlab2.0
CYP2C9(s)	Probability of being CYP substrate	admetlab2.0
CYP2D6(inh)	Probability of being CYP inhibitor	admetlab2.0
CYP2D6(s)	Probability of being CYP substrate	admetlab2.0
CYP3A4(inh)	Probability of being CYP inhibitor	admetlab2.0
CYP3A4(s)	Probability of being CYP substrate	admetlab2.0
Abs	Predicted qualitative human oral absorption: 1, 2, or 3 for low, medium, or high	Qikprop (Schrodinger 2024-4)
OATP1B1(inh)	Probability of being OATP1B1 inhibitor	admetsar2
OATP1B3(inh)	Probability of being OATP1B3 inhibitor	admetsar2
OATP2B1(inh)	Probability of being OATP2B1 inhibitor	admetsar2
%Abs	Predicted human oral absorption on 0 to 100% scale	Qikprop (Schrodinger 2024-4)
Pgp(inh)	Probability of being Pgp-inhibitor	admetlab2.0
Pgp(s)	Probability of being Pgp-substrate	admetlab2.0
M2—descriptors related to atoms (17)		
nAromAt	Number of aromatic atoms	PaDEL-Descriptor v2.21
nAt	Number of atoms	PaDEL-Descriptor v2.21
nAtLAC	Number of atoms in the longest aliphatic chain	PaDEL-Descriptor v2.21
nAtLC	Number of atoms in the largest chain	PaDEL-Descriptor v2.21
nAtPi	Number of atoms in the largest pi system	PaDEL-Descriptor v2.21
nC	Number of carbon atoms	PaDEL-Descriptor v2.21
nCl	Number of chlorine atoms	PaDEL-Descriptor v2.21
nF	Number of fluorine atoms	PaDEL-Descriptor v2.21
nH	Number of hydrogen atoms	PaDEL-Descriptor v2.21
nHBA	Number of hydrogen bond acceptors (using Lipinski’s definition: any nitrogen; any oxygen)	rdkit.Chem.Descriptors
nHBD	Number of hydrogen bond donors (using Lipinski’s definition: Any OH or NH. Each available hydrogen atom is counted as one hydrogen bond donor)	rdkit.Chem.Descriptors
nHeavyAt	Number of heavy atoms (i.e., not hydrogen)	PaDEL-Descriptor v2.21
nN	Number of nitrogen atoms	PaDEL-Descriptor v2.21
nO	Number of oxygen atoms	PaDEL-Descriptor v2.21
nNOCount	Number of nitrogen and oxygen atoms	rdkit.Chem.Descriptors
nX	Number of halogen atoms (F, Cl, Br, I, At, and Uus)	PaDEL-Descriptor v2.21
nHetero	Number of heteroatoms	rdkit.Chem.Descriptors
M3—descriptors related to bonds (10)		
nAromBond	Number of aromatic bonds	PaDEL-Descriptor v2.21
nBonds	Number of bonds (excluding bonds with hydrogen)	PaDEL-Descriptor v2.21
nBonds2	Total number of bonds (including bonds to hydrogens)	PaDEL-Descriptor v2.21
nBondD	Number of double bonds	PaDEL-Descriptor v2.21
nBondM	Total number of bonds that have bond order greater than one (aromatic bonds have bond order 1.5)	PaDEL-Descriptor v2.21
BondS	Number of single bonds (including bonds with hydrogen)	PaDEL-Descriptor v2.21
nRotBond	Number of rotatable bonds, excluding terminal bonds	PaDEL-Descriptor v2.21
nRotBondT	Number of rotatable bonds, including terminal bonds	PaDEL-Descriptor v2.21
RotBondFrac	Fraction of rotatable bonds, excluding terminal bonds	PaDEL-Descriptor v2.21
RotBondTFrac	Fraction of rotatable bonds, including terminal bonds	PaDEL-Descriptor v2.21
M4—charge-based descriptors (23)		
Mac	Mean of absolute charges	chemopy1.0
Mnc	Mean of negative charges	chemopy1.0
Mpc	Mean of positive charges	chemopy1.0
Qass	Sum of squares of charges on all atoms	chemopy1.0
QCmax	Most positive charge on C atom	chemopy1.0
QCmin	Most negative charge on C atom	chemopy1.0
QCss	Sum of squares of charges on C atom	chemopy1.0
QHmax	Most positive charge on H atom	chemopy1.0
QHmin	Most negative charge on H atom	chemopy1.0
QHss	Sum of squares of charges on H atom	chemopy1.0
Qmax	Most positive charge in a molecule	chemopy1.0
Qmin	Most negative charge in a molecule	chemopy1.0
QNmax	Most positive charge on N atom	chemopy1.0
QNmin	Most negative charge on N atom	chemopy1.0
QNss	Sum of squares of charges on N atom	chemopy1.0
Qomax	Most positive charge on O atom	chemopy1.0
Qomin	Most negative charge on O atom	chemopy1.0
QOss	Sum of squares of charges on O atom	chemopy1.0
Rnc	Relative negative charge	chemopy1.0
Rpc	Relative positive charge	chemopy1.0
Tac	Total of absolute charges	chemopy1.0
Tnc	Total of negative charges	chemopy1.0
Tpc	Total of positive charges	chemopy1.0
M5—constitutional descriptors (21)		
fr_aniline	Number of anilines	rdkit.Chem.Descriptors
fr_Ar_N	Number of aromaticnitrogens	rdkit.Chem.Descriptors
fr_benzene	Number of benzene rings	rdkit.Chem.Descriptors
fr_bicyclic	Bicyclic	rdkit.Chem.Descriptors
fr_C_O	Number of carbonyl O	rdkit.Chem.Descriptors
fr_ether	Number of ether oxygens (including phenoxy)	rdkit.Chem.Descriptors
fr_halogen	Number of halogens	rdkit.Chem.Descriptors
fr_NH0	Number of Tertiary amines	rdkit.Chem.Descriptors
FractionCSP3	Fraction of C atoms that are sp3-hybridized	rdkit.Chem.Descriptors
HybRatio	Fraction of sp3 carbons to sp2 carbons	PaDEL-Descriptor v2.21
Mp	Mean atomic polarizabilities (scaled on carbon atom)	PaDEL-Descriptor v2.21
Mpe	Mean atomic Pauling electronegativities (scaled on carbon atom)	PaDEL-Descriptor v2.21
Mv	Mean atomic van der Waals volumes (scaled on carbon atom)	PaDEL-Descriptor v2.21
NHOHCount	Number of NHs or OHs	rdkit.Chem.Descriptors
nAliphCarbocycles	Number of aliphatic (containing at least one non-aromatic bond) carbocycles	rdkit.Chem.Descriptors
nAliphHeterocycles	Number of aliphatic (containing at least one non-aromatic bond) heterocycles	rdkit.Chem.Descriptors
nAromCarbocycles	Number of aromatic carbocycles	rdkit.Chem.Descriptors
nAromHeterocycles	Number of aromatic heterocycles	rdkit.Chem.Descriptors
nSatHeterocycles	Number of saturated heterocycles	rdkit.Chem.Descriptors
Spe	Sum of atomic Pauling electronegativities (scaled on carbon atom)	PaDEL-Descriptor v2.21
Sv	Sum of atomic van der Waals volumes (scaled on carbon atom)	PaDEL-Descriptor v2.21
M6—molecular property descriptors (19)		
AtinRings	Number of atoms in rings	Qikprop (Schrodinger 2024-4)
AMW	Average molecular weight (Molecular weight/Total number of atoms)	PaDEL-Descriptor v2.21
AtPolariz	Sum of the atomic polarizabilities (including implicit hydrogens)	PaDEL-Descriptor v2.21
Hy	Hydrophilic index	chemopy1.0
LogS	Logarithm of aqueous solubility value	admetlab2.0
LogP	Logarithm of partition coefficient	Marvin 20.19
ProtBind	% of protein binding	literature data
MaxRing	Number of atoms in the biggest ring	admetlab2.0
MolReact	Molar refractivity	chemopy1.0
MW	Molecular weight	PaDEL-Descriptor v2.21
nAcid	Number of acidic groups	PaDEL-Descriptor v2.21
nBase	Number of basic groups	PaDEL-Descriptor v2.21
nRigBonds	Number of rigid bonds	admetlab2.0
nStereo	Stereo centers	admetlab2.0
RuleOfFive	Number of violations of Lipinski’s rule of five	Quikprop (Schrodinger 2024-4)
RuleOfThree	Number of violations of Jorgensen’s rule of three	Quikprop (Schrodinger 2024-4)
pKa	Negative base-10 logarithm of acid dissociation constant	admetsar2.0
Polariz	Predicted polarizability in cubic angstroms	Qikprop (Schrodinger 2024-4)
UnsatI	Unsaturation index	chemopy1.0
M7—descriptors related to rings (19)		
n5HeteroRing	Number of 5-membered rings containing heteroatoms (N, O, P, S, or halogens)	PaDEL-Descriptor v2.21
n5Ring	Number of 5-membered rings	PaDEL-Descriptor v2.21
n6HeteroRing	Number of 6-membered rings containing heteroatoms (N, O, P, S, or halogens)	PaDEL-Descriptor v2.21
n6Ring	Number of 6-membered rings	PaDEL-Descriptor v2.21
nF10Ring	Number of 10-membered fused rings	PaDEL-Descriptor v2.21
nFG12HeteroRing	Number of >12-membered fused rings containing heteroatoms (N, O, P, S, or halogens)	PaDEL-Descriptor v2.21
nFG12Ring	Number of >12-membered fused rings	PaDEL-Descriptor v2.21
nFRing	Number of fused rings	PaDEL-Descriptor v2.21
nHeteroRing	Number of rings containing heteroatoms (N, O, P, S, or halogens)	PaDEL-Descriptor v2.21
nRing	Number of rings	PaDEL-Descriptor v2.21
nT10Ring	Number of 10-membered rings (includes counts from fused rings)	PaDEL-Descriptor v2.21
nT5HeteroRing	Number of 5-membered rings (includes counts from fused rings) containing heteroatoms (N, O, P, S, or halogens)	PaDEL-Descriptor v2.21
nT5Ring	Number of 5-membered rings (includes counts from fused rings)	PaDEL-Descriptor v2.21
nT6HeteroRing	Number of 6-membered rings (includes counts from fused rings) containing heteroatoms (N, O, P, S, or halogens)	PaDEL-Descriptor v2.21
nT6Ring	Number of 6-membered rings (includes counts from fused rings)	PaDEL-Descriptor v2.21
nTRing	Number of rings (includes counts from fused rings)	PaDEL-Descriptor v2.21
nAliphRing	Number of aliphatic (containing at least one non-aromatic bond) rings	rdkit.Chem.Descriptors
nAromRing	Number of aromatic rings	rdkit.Chem.Descriptors
nSatRing	Number of saturated rings	rdkit.Chem.Descriptors
M8—topological descriptors (9)		
Arto	Arithmetic topological index by Narumi	chemopy1.0
diametert	Largest value in the distance matrix	chemopy1.0
radiust	Radius based on topology	chemopy1.0
TPSA	Topological polar surface area. Sum of tabulated surface contributions of polar fragments	admetlab2.0
VABC	Van der Waals volume calculated using the method proposed in [Zhao, Yuan H. and Abraham, Michael H. and Zissimos, Andreas M., Fast Calculation of van der Waals Volume as a Sum of Atomic and Bond Contributions and Its Application to Drug Compounds, The Journal of Organic Chemistry, 2003, 68:7368−7373]	PaDEL-Descriptor v2.21
WPATH	Weiner path number	PaDEL-Descriptor v2.21
WPOL	Weiner polarity number	PaDEL-Descriptor v2.21
ZM1	Zagreb index with order 1–2	chemopy1.0
ZM2	Zagreb index with order 1–2	chemopy1.0

**Table A2:** Studies serving as a data source for the PLS model.

Study ID	Reference	Investigated Drugs	Source	Number of Participants	Study Design	Risk of Bias	Gender Specified?	Race Specified?	Meal Type Specified?	Qualitative Composition Specified?	Quantitative Composition Specified?
Chen 2023	[46]	fluconazole	Article	36	non-randomized, open-label, parallel	moderate	yes, males and females	yes	yes	no	yes
Li 2020	[47]	fluconazole	Article	52	non-randomized, open-label, parallel	high	yes, males and females	yes	yes	no	yes
Zimmermann 1994	[48]	fluconazole, itraconazole	Article	12	randomized, open-label, cross-over	moderate	yes, males and females	no	yes	yes	yes
Ahmed 2008	[49]	griseofulvin	Article	9	non-randomized, open-label, longitudinal	high	yes, all males	no	yes	yes	yes
Aoyagi 1982	[50]	griseofulvin	Article	4	non-randomized, open-label, cross-over	high	yes, all males	no	yes	yes	no
None	[51]	ibrexafungerp	SmPC *	NS **	NS	NA #	no	no	yes	no	yes
Schmitt-Hoffmann 2016	[52]	isavuconazole	Article	26	randomized, open-label, cross-over	low	yes, all males	no	yes	no	yes
10850703 study	[53]	itraconazole	Unpublished study	36	randomized, open-label, cross-over	NA	no	no	no	no	no
Barone 1993	[54]	itraconazole	Article	27	randomized, open-label, cross-over	moderate	yes, all males	no	yes	yes	no
Barone 1998	[55]	itraconazole	Article	27	randomized, open-label, cross-over	moderate	yes, all males	yes	yes	yes	no
HGN007	[56]	itraconazole	Unpublished study	NS	non-randomized, open-label, cross-over	high	no	no	yes	no	no
Kapsi 2001	[57]	itraconazole	Article	12	non-randomized, open-label, otherwise not specified	NA	no	no	no	no	no
Lindsay 2018	[58]	itraconazole	Article	20	randomized, open-label, cross-over	moderate	no	no	yes	no	no
MPG017	[59]	itraconazole	Unpublished study	24	randomized, open-label, cross-over	moderate	yes, males and females	no	yes	yes	yes
None	[60]	itraconazole	SmPC	18	randomized, open-label, cross-over	NA	yes, males and females	no	yes	yes	yes
Oguma 2009	[61]	itraconazole	Article	10	non-randomized, open-label, longitudinal	high	yes, all males	yes	yes	yes	no
Rauseo 2021	[62]	itraconazole	Article	52	randomized, open-label, cross-over	moderate	yes, males and females	yes, AA † included	yes	no	yes
Tei 2006	[63]	itraconazole	Article	8	non-randomized, single-blinded, cross-over	high	yes, all males	yes	no	no	no
VandeVelde 2016	[64]	itraconazole	Article	12	randomized, open-label, cross-over	moderate	yes, males and females	no	yes	yes	no
VanPeer 1989	[65]	itraconazole	Article	6	randomized, open-label, cross-over	moderate	yes, all males	no	yes	yes	no
Woo 2008	[66]	itraconazole	Article	8	randomized, open-label, longitudinal	high	yes, all males	yes	yes	yes	yes
Yun 2006-1	[67]	itraconazole	Article	40	randomized, open-label, parallel	moderate	yes, all males	yes	yes	yes	no
Yun 2006-2	[67]	itraconazole	Article	80	randomized, open-label, parallel	moderate	yes, all males	yes	yes	yes	no
Yun 2006-3	[67]	itraconazole	Article	24	randomized, open-label, cross-over	moderate	yes, males and females	yes	yes	yes	no
Daneshmend 1984	[68]	ketoconazole	Article	8	randomized, open-label, cross-over	moderate	yes, males and females	no	yes	yes	yes
Männistö 1982	[69]	ketoconazole	Article	10	randomized, open-label, cross-over	moderate	yes, males and females	no	yes	no	yes
None	[70]	oteseconazole	SmPC	NS	NS	NA	no	no	yes	no	yes
Courtney 2003	[30]	posaconazole	Article	20	randomized, open-label, cross-over	moderate	yes, all males	no	yes	no	yes
Courtney 2004	[71]	posaconazole	Article	12	randomized, open-label, cross-over	moderate	yes, all males	yes, AA included	yes	yes	yes
Dayan 2023	[72]	posaconazole	Article	36	randomized, open-label, cross-over	low	no	no	yes	yes	no
Dogterom 2014-1	[73]	posaconazole	Conference abstract	NS	NS	NA	no	no	yes	no	no
Dogterom 2014-2	[73]	posaconazole	Conference abstract	NS	NS	NA	no	no	yes	no	no
Hens 2016	[74]	posaconazole	Article	5	non-randomized, open-label, cross-over	high	yes, males and females	no	yes	yes	no
Kersemaekers 2015	[75]	posaconazole	Article	18	randomized, open-label, cross-over	high	yes, males and females	yes, AA included	yes	yes	yes
Krishna 2008	[76]	posaconazole	Article	12	randomized, open-label, cross-over	moderate	yes, males and females	yes	yes	no	yes
Krishna 2012	[77]	posaconazole	Article	16	non-randomized, open-label, longitudinal	low	yes, males and females	yes, AA included	yes	no	yes
Li 2019	[78]	posaconazole	Article	18	randomized, open-label, cross-over	low	yes, males and females	yes	yes	yes	yes
Lin 2013	[79]	posaconazole	Article	16	randomized, open-label, cross-over	high	yes, all males	yes	yes	no	yes
None	[80]	posaconazole	SmPC	30	NS	NA	no	no	yes	no	yes
Xu 2013	[81]	posaconazole	Article	12	randomized, open-label, cross-over	moderate	no	yes	yes	yes	yes
Nedelman 1997	[82]	terbinafine	Article	30	randomized, open-label, cross-over	moderate	yes, all males	yes, AA included	no	no	no
None	[83]	voriconazole	SmPC	NS	NS	NA	no	no	yes	no	no
Purkins 2003	[84]	voriconazole	Article	12	randomized, open-label, cross-over	moderate	yes, all males	no	yes	no	yes

* SmPC—summary of product characteristics, ** NS—not specified, # NA—not assessed due to the insufficiently described design, and † AA—African-American.
